# Peanut Rotation and Flooding Induce Rhizobacteriome Variation With Opposing Influences on the Growth and Medicinal Yield of *Corydalis yanhusuo*

**DOI:** 10.3389/fpls.2021.779302

**Published:** 2022-01-07

**Authors:** Xiaodan Li, Songfeng Wang, Yating Fan, Zhe Zhou, Sheng Xu, Penglei Zhou, Jiayu Zhou, Ren Wang

**Affiliations:** ^1^Jiangsu Key Laboratory for the Research and Utilization of Plant Resources, Institute of Botany, Jiangsu Province and Chinese Academy of Sciences, Nanjing, China; ^2^The Jiangsu Provincial Platform for Conservation and Utilization of Agricultural Germplasm, Nanjing, China; ^3^Jiangsu Jiangtong Agricultural Science and Technology Development Co., Ltd., Huaian, China

**Keywords:** *Corydalis yanhusuo*, flooding, medicinal yield, peanut rotation, rhizosphere, 16S rRNA amplicon sequencing

## Abstract

*Corydalis yanhusuo*, a precious herb of the Papaveraceae family, is widely used in multiple traditional Chinese medicines for the treatment of many painful conditions, and its medicinal part is the dried tuber. Yet how to improve this plant’s medicinal yield as well as its economic efficiency remains a key problem in its cultivation. The planting of *C. yanhusuo* in rotation with peanut (*Arachis hypogaea* L.) aims to improve land utilization efficiency, but the total production of tubers is severely reduced relative to fields without rotation. However, an increased yield was observed in *C. yanhusuo* plants grown in previously flooded fields (HR field) compared to the ones grown in the fields that had been used to cultivate peanut (PL field) or in fields without rotation or flooding (N field). Based on these phenomena, in this study, we explored the potential factors responsible for the altered growth/yield of *C. yanhusuo* under different field conditions. Soil physicochemical properties and the diversity and community of rhizobacteriome of *C. yanhusuo* were both analyzed. By testing several soil physicochemical properties, we found that the cation exchange capacity (CEC), soil organic matter (SOM), total nitrogen (TN), and pH value differed significantly among these three types of fields. 16S rRNA amplicon sequencing revealed stark differences in the composition, diversity, and potential functions of the bacterial community in the rhizosphere of *C. yanhusuo* plants grown in field with the peanut rotation or flooding. Notably, the Acidobacteria were enriched in the HR field, while Actinobacteria were enriched in the PL field. More importantly, further analysis showed that changed soil physicochemical properties could be one reason for why the rhizospheric bacterial community has changed; hence, soil physicochemical properties might also be affecting plant performance indirectly by regulating the rhizospheric bacterial community. The RDA analysis distinguished CEC as the most important soil physicochemical property influencing the structure and composition of the *C. yanhusuo* rhizobacteriome. In summary, our results suggest peanut rotation- and flooding-induced soil physicochemical properties changes would further impact the rhizobacteriome of *C. yanhusuo* albeit differentially, culminating in opposite effects upon the plant growth and medicinal yield of *C. yanhusuo*.

## Introduction

*Rhizoma Corydalis* (RC), the dried tubers of the *Corydalis yanhusuo* plant (commonly known as “Yuanhu” in China), is a well-known Chinese herbal medicine that is widely prescribed for treating headaches, spastic and abdominal pains, in addition to relieving pain from injury (Tang and Nie, 2006). Modern medical research has revealed that the *C. yanhusuo* extract has significant inhibitory effects upon acetylcholinesterase (AChE) and cancer cell proliferation, as well as beneficial effects for the cardiovascular system (Ling et al., 2006; Xiao et al., 2011; Zhao Y. et al., 2014; Chen et al., 2016). To date, more than 100 compounds have been isolated and identified from RC, including fatty acids, organic acids, amino acids, sugars, steroids, anthraquinones, volatile compounds, and alkaloids (Hung and Wu, 2016; Abdallah et al., 2017; Hassan et al., 2017). In particular, over 80 types of alkaloids have been isolated and identified (Tian et al., 2020), these being major pharmacologically active compounds for the treatment of blood vessel diseases, tumors, and various pains (Li et al., 2014; Wu et al., 2016). Tetrahydropalmatine is a tetrahydroprotoberberine diisoquinoline alkaloid and the primary active constituent of RC; tetrahydropalmatine has been used as a drug with analgesic, hypnotic, sedative and other effects (Gong et al., 2016; Yu et al., 2016; Liu et al., 2021).

Driven by its large market demand and because of its short planting cycle, many plantations of *C. yanhusuo* have been quickly established for economic gain (Wang Y.M. et al., 2019). The suitable period for planting *C. yanhusuo* is from late September to mid-October, with its harvest in May (Xu, 2005). To make the most use of this land, implementing a crop rotation, generally done with peanut, in the other 4 to 5 months may be economically advantageous. However, to the best of our knowledge, the *C. yanhusuo*-peanut rotation is facing serious problems. After peanut planting, the growth of *C. yanhusuo* plants is severely impaired compared to their cultivation in fields without such a rotation, leading to their reduced medicinal yield. We also found that *C. yanhusuo* plants in flooded fields grew better than those in the monocrop fields without flooding.

We know that the rhizospheric microbiome plays important roles in benefiting plant health and productivity (Philippot et al., 2013; Trivedi et al., 2020), by promoting plants growth, enhancing their nutrient acquisition capacity, and also increasing their resistance to both biotic and abiotic stresses (Maarastawi et al., 2018). Nevertheless, much is still unknown about the rhizospheric microbiome of *C. yanhusuo*. Accordingly, their potential influence on the growth and ensuing medicinal yield of *C. yanhusuo* is also unclear. To fill these knowledge gaps, *C. yanhusuo* plants and rhizospheric soil from three different types of fields were collected and analyzed in this study. For the HR field, during the rainy season, its soil was flooded for 1 month. For the N field, no other plants were planted during the last growing season until *C. yanhusuo* seedlings were planted. In the PL field, *C. yanhusuo* seedlings were planted in the three individual fields that had been used to cultivate peanut in the preceding growing season. Before harvest, both the biomass of *C. yanhusuo* plants and the content of tetrahydropalmatine in their tubers were determined. Further, certain soil physicochemical properties, such as soil moisture, sodium content, and pH value, can strongly influence both the composition and diversity of rhizospheric microbial communities, Peiffer et al. (2013), Edwards et al. (2015), Lüneberg et al. (2018). Therefore, it would be both interesting and timely to explore the interactions among *C. yanhusuo* plants, their rhizospheric bacterial community (rhizobacteriome), and environmental factors.

Given the above documented phenomena in extant fields, this study aimed to assess the changes in soil physicochemical properties and rhizobacteriome in the three different types of *C. yanhusuo* cultivation fields [the N field (mono-crop), the PL field (rotation with peanut), and the HR field (flooded before planting)], to find out the potential soil and microbial factors that could affect the plant growth and yield of *C. yanhusuo*. Our findings provide insight into the complex interactions among medicinal plants, the soil environment, and the rhizospheric microbiome and are useful for guiding the cultivation of *C. yanhusuo* in southeastern China.

## Materials and Methods

### Site Description and Sample Collection

Individuals of *C. yanhusuo* were planted in September, 2020 and collected in April 2021 (i.e., 1 month before harvest). The three field types were located in the Xuyi District (118° 23′ E, 33° 14′ N), Huai’an, Jiangsu Province, China, and consisted of an “HR” field (mono-crop of *C. yanhusuo* that had been flooded in July, 2020), a “PL” field (rotation of peanut and *C. yanhusuo*) and an “N” field (mono-crop of *C. yanhusuo*). Each field type had three replicates, and each replicate was about 300 m^2^. Specifically, for the HR field, *C. yanhusuo* seedlings were planted in three individual fields near the Huai River, and flooded for 1 month in July 2020 for which the submerged water depth was 5–8 cm. For the PL field, *C. yanhusuo* seedlings were planted in three individual fields that were already used to cultivate peanut crops during the prior growing season. For the N field, which served as the control, its three individual fields were left fallow during last growing season (i.e., no crops planted). These three types of fields were adjacent to each other and all managed using the same cultivation practices. These included the application of fertilizer and suitable planting depth and distance as follows: before planting, compound fertilizer (1,050 kg ha^–1^) and urea (45 kg ha^–1^) were applied as base fertilizer in each field (three fields per treatment, nine fields in total); the planting depth was 5–6 cm, and planting distance and row spacing were ∼10 and ∼15 cm, respectively.

Twenty-five individual plants at five positions—the four corners and center—of each replicate field were collected and pooled as a biological replicate sample. Rhizospheric soil samples were collected as described before (Sun et al., 2018; Zuo et al., 2021). Briefly, the roots and tubers were shaken vigorously to remove any loose soil; remaining soils firmly attached to roots and tubers were designated rhizospheric soil, and this material collected with sterile brushes. All samples of rhizospheric soil were then sieved through 2 mm mesh to remove any plant debris. Each soil sample was divided into three portions, with one portion immediately stored at −80°C for DNA extraction followed by 16S rRNA amplicon sequencing; one portion was stored at 4°C to later isolate any culturable bacteria; and another portion was used for the analysis of soil physicochemical properties.

### Extraction and Quantification of Tetrahydropalmatine in *C. yanhusuo*

Four *C. yanhusuo* plants were collected from each field type and each plant served as a biological replicate. Their tubers were oven-dried at 50°C to a constant weight and then ground into powder. From each sample, exactly 0.3 g of powdered tuber was weighed and extracted in 20 mL of a methanol-ammonia solution (20: 1, v/v) for 1 h in an ultrasonic bath at room temperature. Each extraction was filtered through a Millipore filter with a 0.22-μm pore size and stored at 4°C until its high-performance liquid chromatography (HPLC) analysis.

The standard of tetrahydropalmatine (purity ≥ 98%; Shanghai Yuanye Bio-Technology Co., Ltd.) was accurately weighed, dissolved to generate a stock solution at 4.6 mg mL^–1^, and then diluted with methanol into a series of working solutions. For tetrahydropalmatine’s quantification, 20 μL of each sample was injected into an LC-20A HPLC system equipped with an SPD-M20A Photodiode Array Detector (Shimadzu Corporation, Tokyo, Japan). An InertSustain C18 reverse-phase column (5 μm; 200 mm × 4.6 mm I.D.) was used. The optimized parameters were set as follows: the mobile phase consisted of eluent A [0.1% phosphoric acid in water (v/v), adjusted to pH 6.0 with triethylamine] and eluent B (methanol). The flow rate was 0.8 mL min^–1^, and column temperature maintained at 30°C with detections made at 280 nm. The optimal elution program consisted of 0–30 min, using 75% B.

### Soil Physicochemical Properties Measurements

The collected rhizospheric soil was air-dried at room temperature; any visible roots or other debris were manually removed; the soil was then ground and sieved through 2 mm before its characteristics were analyzed. The analyses of different soil physicochemical properties were carried out as described in previous studies. Briefly, soil pH value was measured with a pH-meter using a soil suspension (soil: 0.01 M CaCl_2_ = 1:2.5, w/v) (Lu, 2000). Soil organic matter (SOM) was determined using the K_2_CrO_7_ oxidation titration method, as described by Lu (2000). Soil total nitrogen (TN) was measured with an element analyzer according to Kjedahl method (Lu, 2000). Soil was digested by H_2_SO_4_/HClO_4_ and total phosphorus (TP) then determined by the molybdenum blue method. Total potassium (TK) in soil was quantified using flame photometry after its aqua-regia digestion (Xiao et al., 2015). Soil cation exchange capacity (CEC) was determined by the exchange with ammonium acetate (NH_4_OAc, 1.0 M, pH 7.0) and titration with 0.05 M HCl (Dai et al., 1998). Soil texture—percentages of sand, silt, and clay components—was determined by a laser particle size analyzer (Master-Sizer 3000, Malvern Panalytical, Malvern, United Kingdom) according to the Stokes law, for which classification names were assigned following the “textural triangle” developed by the USDA (Zhao, 1981).

### Culturable Bacterial Isolation and Identification

Soil samples were resuspended and serially diluted in sterile water and plated on nutrient agar (NA) plates. All plates were incubated at 30°C for 48–60 h, after which the number of colony-forming units (CFUs) was counted per plate. Culturable bacteria concentrations were calculated and expressed as CFU per gram soil (CFU g^–1^ soil). Next, every single visible bacterial colony was picked, purified on fresh NA medium, and stored in 15% glycerol at –80°C. From each, its total DNA was extracted using the TIANamp Bacteria DNA Kit (Tiangen Biotech Co., Ltd., Beijing, China) according to manufacturer’s protocol. The primer pair of 27F (5′-AGAGTTTGATCCTGGCTCA-3′) and 1492R (5′-GGTTACCTTGTTACGACTT-3′) was used to amplify the 16S rRNA gene. The sequence of each bacterial colony was BLASTed against the NCBI database and identified accordingly.

### Rhizospheric Bacterial DNA Extraction, PCR Amplification, 16S rRNA Amplicon Sequencing Processing, and Data Analysis

Rhizospheric bacterial DNA was extracted using the HiPure Soil DNA Kit (Guangzhou Magen Biotechnology Co., Ltd., Guangzhou, China) and following the manufacturer’s protocols. The 16S rDNA V3-V4 region was amplified by PCR, using the primers 341F (5′-CCTACGGGNGGCWGCAG-3′) and 806R (5′-GGACTACHVGGGTATCTAAT-3′). The ensuing PCR amplification product was recovered by using an E.Z.N.A. Gel Extraction Kit (Omega bio-tek Inc., GA, United States), for which Illumina Misequence PE 250 (Beijing Allwegene Biotechnology Co., Ltd., Beijing, China) was then used for its sequencing. Raw sequences were deposited in the SRA database under the accession number PRJNA762442. Based on their distribution, nearly all the tags were within the length range of 440–470 bp and rather high in quality. To avoid the bias from different depths of sequencing for the alpha and beta diversity measurements, all libraries were normalized to the same size (104,909 tags in PL-2), based on the library with the lowest number of sequences.

Raw reads were filtered using fastp (v 0.18.0) to remove any reads containing more than 10% unknown nucleotides and reads which contained less than 50% of bases with quality values (Q values) above 20 (Chen et al., 2018). Paired end clean reads were merged as raw tags by using the FLASH tool (v 1.2.11) with a minimum overlap of 10 bp and a mismatch error rate of 2% (Magoè and Salzberg, 2011). Noisy sequences of raw tags were filtered under specific filtering conditions (Bokulich et al., 2013). The clean tags were clustered into operational taxonomic units (OTUs) of ≥ 97% similarity using the UPARSE (v 9.2.64) pipeline (Edgar, 2013). All chimeric tags were removed using the UCHIME algorithm (Edgar et al., 2011). The representative OTU sequences were classified by a naïve Bayesian model using the RDP classifier (v 2.2) (Wang et al., 2007) based on SILVA database (v 132) (Pruesse et al., 2007), with the confidence threshold value of 0.8. Venn analysis was performed using the R project VennDiagram package (v 1.6.16) to identify unique and common OTUs in each group (Chen and Boutros, 2011). Circular layout representations of species abundance were graphed using Circos (v 0.69-3) (Krzywinski et al., 2009). Taxonomic composition of bacteria that are present in different samples was visualized using a stack bar plot at different taxonomic level using ggplot2 package (v 2.2.1) (Wickham, 2011). Alpha diversity indexes and species richness estimators in each group was evaluated in QIIME (v 1.9.1) (Caporaso et al., 2010). Phylogenetic diversity-whole tree (PD tree) was calculated in Picante (v 1.8.2) (Kembel et al., 2010). PCoA (principal coordinates analysis) of (Un) weighted unifrac was generated in the R project Vegan package (v 2.5.3) (Oksanen et al., 2010) and plotted in the R project ggplot2 package (v 2.2.1) (Wickham, 2011). Biomarker features in each group were screened by LEfSe software (v 1.0) (Segata et al., 2011). The KEGG pathway analysis of the OTUs was inferred using Tax4Fun (v 1.0) (Aßhauer et al., 2015). Analysis of function difference between groups was calculated by Welch’s *t*-test in the R project Vegan package (v 2.5.3) (Oksanen et al., 2010).

Redundancy analysis (RDA) was implemented to test whether and which soil physicochemical properties contributed significantly (*p* < 0.05) to variation in the bacterial community of the *C. yanhusuo* rhizosphere. RDA and Mantel test was executed in R project Vegan package (v 2.5.3) (Oksanen et al., 2010) to clarify the influence of environmental factors on community composition of *C. yanhusuo* rhizosphere. All these analyses of 16S rRNA amplicon sequencing data and according figures were generated using Omicsmart^1^, a dynamic real-time interactive online platform for data analysis (He et al., 2021).

### Statistical Analysis

Analyses were carried out using SPSS 13.0 (SPSS Inc., Chicago, IL, United States) and Microsoft Excel 2019 software programs. A threshold of *p* < 0.05 was used to define statistically significant results.

## Results

### Contrasting Effects of Peanut Rotation and Flooding on the Yield of *C. yanhusuo*

**FIGURE 1:**
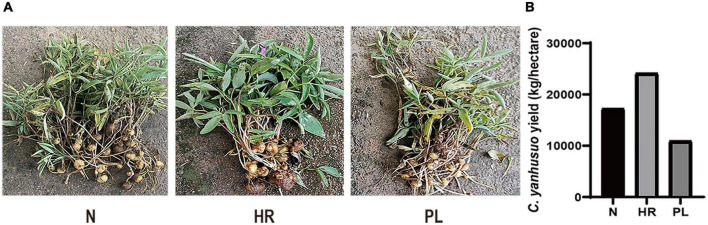
The effect of different field types upon the *Corydalis yanhusuo* tuber yield. **(A)** Photographs of *C. yanhusuo* plants from the three different fields. **(B)** The total tuber yield per hectare of *C. yanhusuo* tuber from each field type. PL, plants from the PL field (field under peanut–*C. yanhusuo* rotation); N, plants from the N field (*C. yanhusuo* field without rotation and flooding); HR, plants from the HR field (*C. yanhusuo* field that had been flooded in July, 2020).

In this study, *C. yanhusuo* plants of the same age but under different field cultivation conditions (HR, PL, and N) were harvested. The *C. yanhusuo* plants in the N and HR fields were healthy and their tubers were big, plump, and solid, but those plants in the PL field were small and appeared feeble (Figure 1A). Moreover, compared with those of N or HR plants, the tubers of PL plants were significantly smaller which directly diminished their yield as the medicinal part (Figure 1B). Accordingly, the total yield of *C. yanhusuo* tubers from the HR field was at least 24,150 kg ha^–1^, while that from the N and PL fields, respectively, was 17,250 and 10,950 kg ha^–1^ (Figure 1B). These results indicated that flooding could promote the growth of *C. yanhusuo* and its ensuing medicinal yield, whereas its rotation with peanut could be disadvantageous. Further, there was no significant difference in the content of tetrahydropalmatine in *C. yanhusuo* plants among the three field types (Supplementary Table 1). Building on this result, we next explored the mechanisms underlying the disparities in plant performance under the different cultivation conditions.

### Altered Soil Physicochemical Properties Among the Three Different Fields

The TN in soil was significantly higher at PL than the other two fields (Table 1), and both N and PL fields harbored greater amount of SOM than HR did (Table 1). By contrast, the TP was similar among all soils, as was TK (Table 1).

**TABLE 1:** Physicochemical properties of soils from three different cultivation fields of *C. yanhusuo* in the Xuyi District, eastern China.

Sample	pH	CEC (cmol kg^–1^)	TN (g kg^–1^)	SOM (g kg^–1^)	TP (g kg^–1^)	TK (g kg^–1^)
N	8.43 ± 0.06 ab	7.81 ± 0.34 c	0.88 ± 0.01 b	14.26 ± 0.63 a	1.46 ± 0.08 a	17.76 ± 0.72 a
HR	8.50 ± 0 a	10.41 ± 0.36 a	0.89 ± 0.02 b	11.77 ± 0.79 b	1.34 ± 0.05 a	17.63 ± 0.42 a
PL	8.4 ± 0 b	9.18 ± 0.34 b	1.09 ± 0.06 a	15.12 ± 0.60 a	1.31 ± 0.08 a	16.88 ± 0.32 a

*CEC, cation exchange capacity; TN, total nitrogen; SOM, soil organic matter; TP total phosphate; TK, total potassium.*

*The pH, CEC, TN, SOM, TP, and TK values (means ± SD, n = 3) with different lower-case letters (a–c) are significantly different (p < 0.05).*

*N, soil from the C. yanhusuo field without rotation; HR, soil from the C. yanhusuo field that had been flooded in July 2020; PL, soil from the field under peanut–C. yanhusuo rotation.*

Soil pH values were significantly different between the HR and PL fields (*p* < 0.05) (Table 1). Compared with the N field, the soil pH in the HR and PL fields were 0.83% higher and 0.36% lower, respectively. The strongest effect sizes were found for the CEC of different soils, which were ranked as follows: HR > PL > N (Table 1). Although changes to soil physicochemical properties across the fields were detected, the direct influences of soil characteristics cannot fully explain the changed plant growth and tuber yields of *C. yanhusuo*.

### Influence of Peanut Rotation and Flooding on *C. yanhusuo* Rhizospheric Bacterial Composition and Diversity

The concentration of culturable bacteria evidently varied among the different soils (Supplementary Figure 1). Compared with soil from the N (29.2–38.1 × 10^7^ CFU g^–1^ soil) and HR (24–27.5 × 10^7^CFU g^–1^ soil) fields, the bacterial CFU number in soil of the PL field (approximately 44.1–39.9 × 10^7^ CFU g^–1^ soil) was significantly higher (*p* < 0.05). Hence, the inhibition of plant growth observed in the PL field might have arisen from the enrichment of some harmful bacteria in its rhizospheric soil. To gauge and understand which bacterial taxa were enriched and depleted in the different soils, each bacterial strain was identified based on 16S rDNA sequencing.

From all soil samples, a total of 194 strains were identified that belonged to 61 genera. *Bacillus* (24%), *Arthrobacter* (8%) and *Microbacterium* (6.7%) were the most abundant genera in the PL soil (Supplementary Figure 2). Although *Bacillus* (16.1%), *Arthrobacter* (17.7%), and *Microbacterium* (8.1%) were the most abundant bacterial genera in the N soil as well, their relative proportions differed considerably from those in PL soil. The top three genera present in HR soil were *Bacillus* (10.6%), *Arthrobacter* (10.6%), and *Streptosporangium* (8.5%). Therefore, these compositional changes in the rhizobacteriome might explain the altered performance of *C. yanhusuo* across the three field types.

**FIGURE 2:**
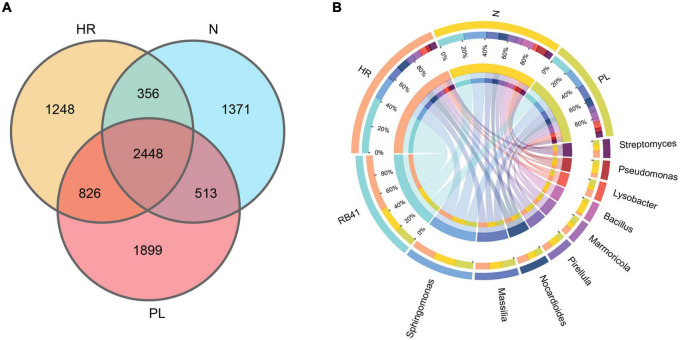
Venn diagram of rhizosphere-enriched bacterial OTUs in rhizospheric soils from the three field types. **(A)** Venn diagram representing the bacterial OTUs associated with the rhizosphere of *C. yanhusuo*. **(B)** CIRCOS diagram illustrating the taxonomic representation of differences between N, HR and PL fields. The width of each ribbon represents the abundance of each taxon. N, soil from the *C. yanhusuo* field without rotation; HR, soil from the *C. yanhusuo* field that had been flooded in July 2020; PL, soil from the field under peanut–*C. yanhusuo* rotation.

The bacterial community in the rhizosphere of *C. yanhusuo* plants grown in each field type was further characterized by 16S rRNA amplicon sequencing, whose results corroborated the changed rhizospheric bacteriome community structure of plants growing in different soils across the fields. The number of OTUs varied from 5,497 to 6,428 per sample (mean = 5,890) (Supplementary Table 2). The number of bacterial OTUs differed significantly in abundance among the different rhizospheric soils. As the Venn diagram shows (Figure 2A), overall, 2,448 OTUs were detected in the rhizosphere of plants grown in all three fields’ soil. Notably, plants grown in PL soil harbored 1,899 unique OTUs, far more that in plants cultivated in the N (1,371 OTUs) and HR (1,248 OTUs) soils. Circos plots (Figure 2B) revealed that samples from N and PL soils were associated with RB41 (ca. 20%) and *Sphingomonas* (ca. 20%), while their association with *Massilia* (< 10%) and *Nocardioides* (<10%) appeared less pronounced. Rhizospheric soil samples from the HR field sustained a higher abundance of RB41 (ca. 40%) in comparison with the N and PL fields. Despite sharing a similar microbial community composition, evidently there were some key differences in the abundance of certain genera among the rhizospheric soil samples from different cultivation fields. Circos plots also showed that the PL field presented a higher association with *Massilia* (>15%) (Figure 2B). Additionally, *Pseudomonas* seemed to be more abundant in the N field (Figure 2B).

**FIGURE 3:**
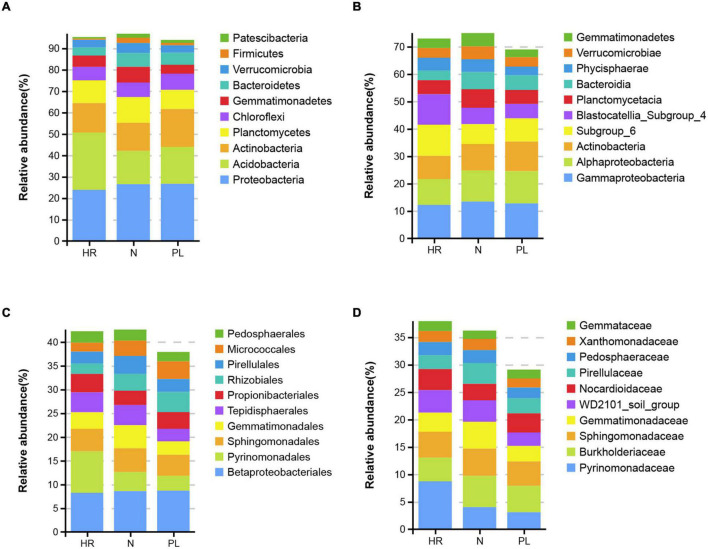
Top 10 relative abundances of bacterial communities classified at the phylum **(A)**, class **(B)**, order **(C)**, and family **(D)** levels in different rhizospheric soils. N, soil from the *C. yanhusuo* field without rotation; HR, soil from the *C. yanhusuo* field that had been flooded in July 2020; PL, soil from the field under peanut–*C. yanhusuo* rotation.

The most abundant bacteria at different taxonomic levels in three fields are presented in Figure 3. Acidobacteria, Proteobacteria, and Actinobacteria were the most abundant phyla in all the soils (Figure 3A), but the most abundant phylum differed among the field types. The relative abundance of Acidobacteria was highest in HR field (26.8%) followed by PL (17.3%) and N (15.5%) fields. The relative abundance of Proteobacteria (26.7%) was greatest in PL field, followed by N (26.5%) and HR (23.9%) fields; PL field also possessed the highest relative abundance of Actinobacteria (17.7%), whose abundance was lower in the two other fields: HR (13.7%) and N (13.2%). At the class level, the relative abundances of Alphaproteobacteria and Bacteroidia in HR field were low, while Subgroup_6 and Blastocatellia_Subgroup_4 were significantly more abundant in HR field (Figure 3B). At the order level (Figure 3C), the relative abundance of Pyrinomonadales in the N (4%) or PL (3.1%) field was low, while the relative abundance in HR (8.7%) was significantly higher. Betaproteobacteriales (belonging to Gammaproteobacteria) was the most abundant order. Gemmatimonadales was relatively lower in abundance in PL (2.8%) than that in either HR (3.5%) or N (4.9%). For both Rhizobiales and Micrococcales, the relative abundance of each was lower in HR field. Yet at the family level, Pyrinomonadaceae was most abundant in HR (8.7%), while in N and PL, its relative abundance was 4.0 and 3.1%, respectively (Figure 3D).

**FIGURE 4:**
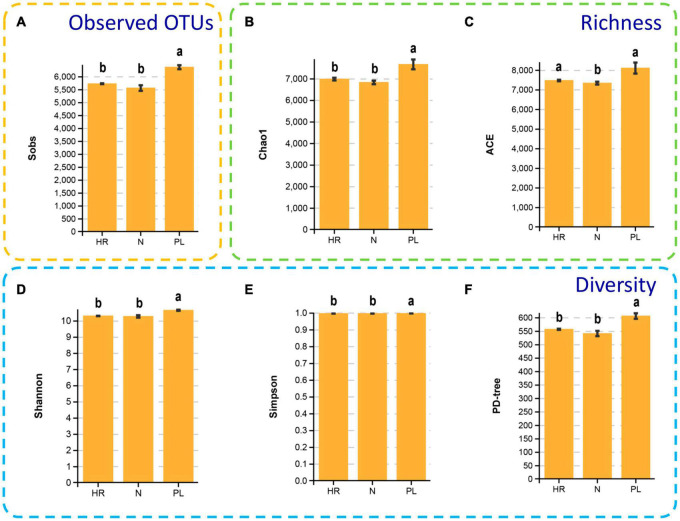
Alpha diversity of the microbial communities of the rhizospheric bacterial communities of the N, HR, and PL field samples. Sobs **(A)**, Chao1 **(B)**, ACE **(C)**, Shannon **(D)**, Simpson **(E)**, and PD-tree **(F)**. Different letters indicate significant differences (Tukey-HSD, *p* < 0.05). N, soil from the *C. yanhusuo* field without rotation; HR, soil from the *C. yanhusuo* field that had been flooded in July 2020; PL, soil from the field under peanut–*C. yanhusuo* rotation.

It is noteworthy that the α diversity indexes (Shannon, and Simpson), diversity estimator (PD-tree), species richness estimators (ACE and Chao 1), the observed species (sobs) all showed a similar pattern in different fields (Figure 4). The rarefaction curve showing Good’s coverage appears in Supplementary Figure 3. The rhizospheric microbiome of the PL field had significantly higher α diversity in comparison to the other two fields (N and HR); correspondingly, species richness estimators (ACE and Chao 1) were lower in the N and HR microbiomes. The Shannon index of PL was 10.66 ± 0.03, being significantly higher than that of N (10.28 ± 0.07) and HR (10.31 ± 0.01) (Figure 4D). Moreover, Simpson index, PD-tree, and the species richness estimators including Chao 1, ACE, all showed similar trends (Figure 4). Taken together, these results indicated the composition and diversity of the bacterial community in the rhizosphere of *C. yanhusuo* varied non-randomly among the tested soil samples. Evidently, using a peanut rotation could increase the α diversity of rhizobacteriome. Still, how the presence of more diverse bacteria in the rhizosphere might be linked to a negative influence on plant performance needs further explanation.

**FIGURE 5:**
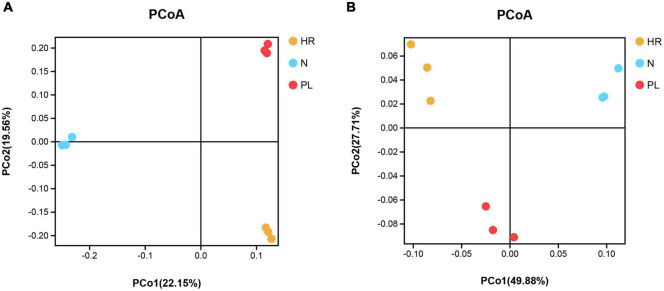
Principal coordinates analysis (PCoA) based on the unweighted UniFrac (UU) and weighted UniFrac (WU) distances among different rhizospheric soil bacterial communities of β-diversity: **(A)** UU distance; **(B)** WU distance. Symbols with unique colors denote different samples: yellow circle for HR, soil from the *C. yanhusuo* field that had been flooded in July 2020; blue circle for N, soil from the *C. yanhusuo* field without rotation; red circle for PL, soil from the field under peanut–*C. yanhusuo* rotation).

Samples from the different fields were clearly separated in the pooled principal coordinates analysis (PCoA) at the OTU level, whether based on unweighted UniFrac (UU) or weighted UniFrac (WU) distances (Figure 5). This suggested that flooding or a peanut rotation could significantly and differentially influence the rhizospheric bacterial diversity of *C. yanhusuo*. Using the UU distance, a distinct pattern was discernible for the rhizospheric bacterial communities. The two axes explained 22.15 and 19.56% of the total variation in rhizobacteriome (Figure 5A); however, when based on the WU distance, the first and second axes were now able to respectively explain 49.88 and 27.71% of that variation (Figure 5B).

### Effects of Environmental Factors on Microbial Community

**FIGURE 6:**
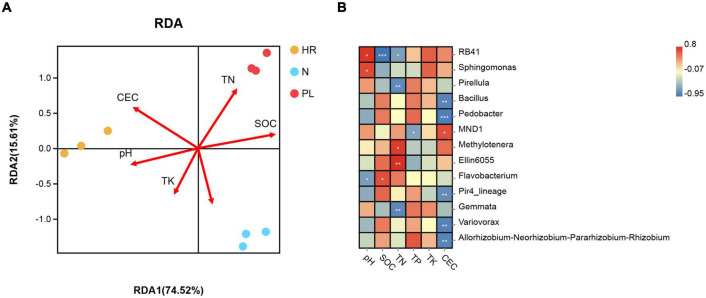
Relationships between microbial community and soil properties **(A)** Redundancy analysis (RDA) of soil properties and bacterial abundance in the *C. yanhusuo* rhizosphere. The angle and length of the arrows indicate the direction and strength of the relationships of soil factors vis-à-vis the microbial populations. CEC, cation exchange capacity, SOM, soil organic matter, TN, total nitrogen, TP, total phosphorus, TK, total K. The CEC (*r*^2^ = 0.798, *p* = 0.002), SOM (*r*^2^ = 0.382, *p* = 0.036) and TP (*r*^2^ = 0.308, *p* = 0.047) were significant factors that influenced the microbial community structure of the *C. yanhusuo* rhizosphere. **(B)** Genus influenced by different soil properties in the rhizosphere of *C. yanhusuo* (*p < 0.05, **p < 0.01, ***p < 0.001; Spearman correlations). N, soil from the *C. yanhusuo* field without rotation; HR, soil from the *C. yanhusuo* field that had been flooded in July 2020; PL, soil from the field under peanut–*C. yanhusuo* rotation.

Redundancy analysis (RDA) let us evaluate whether the changed soil physicochemical properties were paramount driver of the varied rhizospheric bacterial community among the three different fields (Figure 6A). When applied to the main bacterial genera, the RDA showed that the first two axes accounted for 90.13% of the total variance in bacterial community composition, with the first axis capturing 74.52% of that variance. Among all the environmental factors examined using the Mantel test, CEC (*r* = 0.798, *p* = 0.002), SOM (*r* = 0.382, *p* = 0.036), and TP (*r* = 0.308, *p* = 0.047) were significantly correlated with microbial community structure at the genus level (Supplementary Figures 4B,D,F), whereas the other soil factors were negligibly correlated with it (*p* > 0.05) (Supplementary Figures 4A,C,E). These results indicated that CEC was the most critical soil factor potentially shaping the structure of rhizosphere microbial communities. At the genus level, CEC were associated with an excess of MND1, and significantly associated with less of *Bacillus*, *Pedobacter*, Pir4_lineage, *Variovorax*, and *Allorhizobium*–*Neorhizobium*–*Pararhizobium*–*Rhizobium* (Figure 6B); hence, these bacterial taxa could have potential influence on the growth of *C. yanhusuo*.

### Indicator Species Analysis and Bacterial Functions’ Prediction in the Rhizospheric Community of *C. yanhusuo* Grown in Different Fields

**FIGURE 7:**
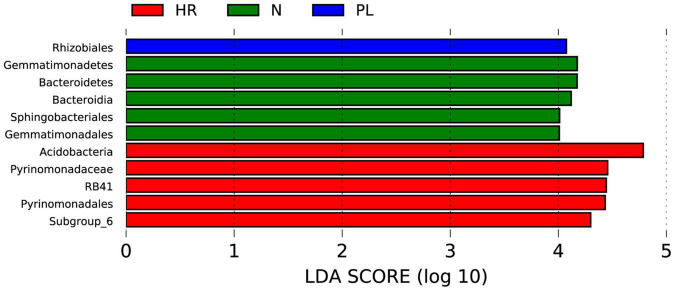
Linear discriminant analysis Effect Size (LEfSe) analysis of microbial abundance in the HR, N, and PL fields. The histogram of linear discriminant analysis (LDA) scores computed for differentially abundant microbes among different samples (these identified at a threshold value of 4.0). N, soil from the *C. yanhusuo* field without rotation; HR, soil from the *C. yanhusuo* field that had been flooded in July 2020; PL, soil from field under peanut–*C. yanhusuo* rotation.

To identify biomarkers in the rhizosphere of each field type, a linear discriminant analysis effect size (LEfSe) analysis was carried out (Figure 7). We found fewer biomarkers for the PL than the N and HR groups. The LEfSe analysis—using a linear discriminant analysis (LDA) score threshold of 4.0, at *p* < 0.05—showed that the Rhizobiales occurred only in PL soil (Figure 7), which must somehow be related to its peanut rotation. Logically then, the bacteria belonging to Rhizobiales could not have had a beneficial effect upon *C. yanhusuo* plants. Bacteroidetes, Bacteroidia, Sphingobacteriales, Gemmatimonadetes, and Gemmatimonadales were biomarkers in the N group, while Acidobacteria, Pyrinomonadaceae, RB41, Pyrinomonadales, and Subgroup_6 were biomarkers in the HR group (Figure 7).

To further assess how changed soil properties might have influenced the functional profiles of rhizospheric bacterial communities in the three different fields, a Tax4Fun analysis was carried out to predict bacterial functioning (Wang X.J. et al., 2020). As seen in Supplementary Figure 5A, more metabolic pathways of bacterial communities in the N and HR fields emerged at level 2, including amino acid metabolism, biosynthesis of other secondary metabolites, replication and repair, signal transduction, glycan biosynthesis and metabolism, cell motility, and translation pathways, among others, but the opposite pattern characterized the PL soil. These prediction results demonstrated that the potential bacterial functions shifted drastically among the N, HR and PL soil samples. The Tax4Fun-predicted significant abundances of carbohydrate metabolism in PL soil when compared with N soil or HR soil (*p* < 0.05, Welch’s *t*-test) (Supplementary Figures 5B,D). This suggested that carbohydrate metabolism might play a role in the adaptation of rhizospheric bacteria in the soil with *C. yanhusuo*-peanut rotation.

## Discussion

When *C. yanhusuo* plants were grown in the PL field, we found the yield of their tubers and thus medicinal parts were severely decreased when compared to those harvested from plants cultivated in the N field. By contrast, enhanced growth of the *C. yanhusuo* plants occurred in the HR field, a phenomenon common in southeast China. To explore why disparities in plant growth and tuber yield were observed under the different field conditions, firstly, local soil physicochemical properties were measured, some of which could directly influence plant growth while may indirectly influence by regulating the rhizospheric bacterial community of *C. yanhusuo*. Secondly, we investigated the bacterial community in the rhizosphere of *C. yanhusuo* plants cultivated in the different fields. This latter analysis showed significantly higher bacterial diversity in rhizosphere of *C. yanhusuo* plants from the PL field than the other two fields (Figure 4), with Rhizobiales exclusively enriched in the PL field (Figure 7). It is plausible that the enrichment of Rhizobiales might lead its members to occupy too much of an ecologic niche shared with other bacterial taxa in the rhizosphere, thus limit the growth of other plant-growth promoting microbes. Moreover, the changed soil physicochemical properties were found capable of affecting the composition and diversity of the rhizospheric bacterial community (Figure 6). Thus, this study showed that peanut rotation and flooding induced different alterations in soil physicochemical properties, some of which could further differentially affect the rhizospheric microbiome of *C. yanhusuo*. Given that the rhizospheric microbiome is known to play an important role in shaping plant phenotypes (Panke-Buisse et al., 2015), its modification likely underpinned the opposite responses observed in plant growth and medicinal yield of *C. yanhusuo* in the PL and HR fields.

Soil is one of the richest microbial ecosystems on Earth and it serves as a reservoir for myriad colonizers that form plant root-associated microbiomes (Zuo et al., 2021). A previous study estimated that there were more than 2,000 bacterial species in 0.5 g of soil (Wagg et al., 2014). Rhizospheric microbes have close relationships with the roots of plants, colonizing their surface or even their interior; these microbes could benefit plant adaptation and growth in various ways, mainly by secreting hormones, dissolving plant inaccessible nutrients, and resisting phytopathogens (Wang X. et al., 2020). Moreover, it was recently concluded that soil factors are key determinants influencing the rhizobacteriome in multiple stressed environments, including those prone to flooding (Thomas-Barry et al., 2021). Thus, it is reasonable to suppose the contrasting plant performances observed in the PL and HR fields were due to a modified rhizobacteriome as induced by the peanut rotation and flooding, respectively. In recent research, flooding stress was suggested as a factor that may increase bacterial activity and energy metabolism (Mavrodi et al., 2018; Liu et al., 2020), while rotation of crops can differentially impact rhizosphere microbial communities (Peng et al., 2016). Hence, here we speculated that the peanut rotation and flooding each induced specific changes in soil physicochemical properties and then, accordingly, in the rhizobacteriome, whose modification further influenced the growth of *C. yanhusuo*.

A variety of soil characteristics like clay content, organic carbon content, pH value, water holding capacity, and so forth have been reported to shape the soil microbial community (Zhao J. et al., 2014; Bai et al., 2017). Our results suggest the soils in all the three field types were sandy loam soils according to the soil texture triangle technique developed by the USDA (Supplementary Table 3). We also found that the low SOM content (11.77 ± 0.79 g kg^–1^) and high CEC (10.41 ± 0.36 cmol kg^–1^) of the HR soil might indirectly benefit the growth of *C. yanhusuo* vis-à-vis the other two soils (Table 1). The SOM is determined by the dynamic balance between microbial metabolism and plants’ carbon input (Janzen, 2015). Accordingly, we observed that HR soil had lower bacterial abundance, which might explain its low SOM. Arguably, the interactions between soil properties and rhizospheric bacterial community are bidirectional and complex. The PL soil had a high SOM, TN, and microbial abundance due to the peanut rotation. A significant positive correlation between CEC and bacterial species distribution was observed (Supplementary Figure 4), which implied that variables associated with CEC may be crucial determinants of bacterial community structure in the *C. yanhusuo* rhizosphere. Recent work has proven that CEC is relatively higher in soils featuring a higher buffering capacity against acidification (Shu et al., 2019; Thomas-Barry et al., 2021), and this is significantly correlated with rhizobacteriome community assembly and bacterial functioning (de Carvalho Mendes et al., 2012). Thus, our findings agree with those of a previous study which suggested CEC is a common global variable for explaining microbial community structure across different terrestrial biomes (Docherty et al., 2015). Moreover, SOM and CEC were each greatest in HR soil, indicating that flooding might have positive effects on both edaphic factors. Moreover, Bastida et al. (2017) reported soil water was a key factor that could impact the bacterial community, which is consistent with our study’s results.

The rhizosphere zone serves as a vital interface for plant-microbe interactions and many physiological and ecological processes occur there, which are essential for plants’ adaption, growth, and development (Mendes et al., 2014; Sohn et al., 2021). Microbes have faster adaptive responses to changing edaphoclimatic conditions and could thereby gain competitive evolutionary advantages because of their shorter life cycles (Waller et al., 2005). As such, the composition and diversity of bacterial community in the rhizosphere of *C. yanhusuo* was quickly modified in response to the peanut rotation. Specifically, the Sobs, Chao1 (species richness estimators) along with Simpson and Shannon indexes, all increased in PL soil. Another study also reported on changes in the soil bacterial community associated with plant growth and development in response to crop rotation (Panke-Buisse et al., 2015). We also found that the OTU number and Shannon diversity index was much higher for rhizospheric communities of *C. yanhusuo* than of other medicinal plants, or even peanut (Gao et al., 2019; Wang X.L. et al., 2019). The OTUs and Shannon diversity index of rhizospheric soil would be jointly affected by multiple factors (cultivation site, season, rainfall, soil type, etc.), hence, we speculate that some specific factors existing in the rhizosphere of *C. yanhusuo* fostered the higher OTU number and Shannon index observed in this study, a view that concurs with another study concerning a high OTU number and Shannon index in bacterial community in the rhizosphere of *C. yanhusuo* grown under different environmental conditions (Li et al., 2019).

Acidobacteria and Proteobacteria are two dominant phyla present in many soil environments (Clark et al., 2009; Prober et al., 2015). According to our results, the dominant bacterial phyla (i.e., relative abundance > 3%) in the rhizosphere of *C. yanhusuo* cultivated in the soils from all three field types were Proteobacteria, Acidobacteria, Actinobacteria, Bacteroidetes, Planctomycetes, Chloroflexi, Gemmatimonadetes, Bacteroidetes, and Verrucomicrobia (Figure 3A). It has been shown that bacteria could rapidly adapt to abiotic stresses (heavy metal pollution or flooding), possibly due to their faster metabolism and more extensive substrate utilization (Unger et al., 2009). In our study, three major groups at the phylum level (Proteobacteria, Acidobacteria, and Actinobacteria) were dominant in the bacterial community. Other work suggested the phylum Proteobacteria is prevalent in wetland soils (Ligi et al., 2014). Although the bacterial communities in the rhizosphere of *C. yanhusuo* grown in the three field types did differ in their abundance of rhizospheric bacterial taxa, we found they were nonetheless concentrated in Proteobacteria, Acidobacteria, and Actinobacteria (Figure 3A). The composition of rhizospheric bacteria communities was not constant among *C. yanhusuo* plants grown in the different fields. Moreover, the percentage of Acidobacteria decreased in N and PL soils compared to HR soil. Acidobacteria, which are widespread and thrive in nutrient-poor soil, are reportedly able to reduce levels of nitrates and nitrites (Schmidt, 2019). We used the indicator value (IndVal) coefficient analysis developed by Dufrêne and Legendre (1997) to identify the bacterial taxa that significantly contributed to differences among the three field types. According to the indicator value, both Pyrinomonadaceae and Rhodanobacteraceae were significantly abundant in HR soil (Supplementary Figure 6). In a recent study, Pyrinomonadaceae exerted a strong positive influence upon plant development (Obermeier et al., 2020). Thus, we speculate the enrichment of some beneficial bacterial taxa is what fostered better plant growth in the HR field. Furthermore, we are currently conducting studies that focus on screening specific bacteria isolated from the rhizosphere of *C. yanhusuo* plants cultivated in different fields. Compared with culture-independent (NGS) data, we found that 19.1% of bacterial genera were isolated. This isolation rate is admittedly not high, which could be due to limitations of the isolation method we used. In future research steps planned, we will improve our isolation methods to isolate more bacteria, including anaerobic/microaerophilic bacteria, which escaped detection and thus identification in this study. That work aims to further elucidate and explain the complex mechanistic interactions among the soil environment, rhizospheric microbiome, and plant growth.

Overall, this study reveals that the composition and diversity of the bacterial community dwelling in the rhizosphere of *C. yanhusuo* is significantly influenced by peanut rotation and flooding, which can further affect this plant’s performance and its eventual medicinal yield. Our results also illustrate how the influences of peanut rotation and flooding upon *C. yanhusuo* rhizospheric bacterial communities are related to changed soil properties. The soil micro-ecological environment and soil nutrient imbalance may lead to serious declines in the biomass of *C. yanhusuo* grown in fields with a peanut rotation. In summary, this study has helped to fill knowledge gaps in the rhizospheric bacterial community of *C. yanhusuo* and it provides a basis for the subsequent mining of microbial functions. Moreover, the findings advance our understanding of rotation effects in *C. yanhusuo*’s cultivation and could prove useful for developing promising strategies to improve its medicinal yield and thereby enhance its economic benefit to farmers.

## Data Availability Statement

The datasets presented in this study can be found in online repositories. The names of the repository/repositories and accession number(s) can be found below: https://www.ncbi.nlm.nih.gov/, PRJNA762442.

## Author Contributions

RW designed the study. XL and PZ performed the sample collection. YF performed the bacteria isolation and identification. SW performed the soil characters measurements. SX and ZZ performed the tetrahydropalmatine content determination. XL performed the statistical analyses and manuscript preparation. JZ revised the manuscript. All authors contributed to the article and approved the submitted version.

## Conflict of Interest

PZ was employed by the company Jiangsu Jiangtong Agricultural Science and Technology Development. The remaining authors declare that the research was conducted in the absence of any commercial or financial relationships that could be construed as a potential conflict of interest.

## Publisher’s Note

All claims expressed in this article are solely those of the authors and do not necessarily represent those of their affiliated organizations, or those of the publisher, the editors and the reviewers. Any product that may be evaluated in this article, or claim that may be made by its manufacturer, is not guaranteed or endorsed by the publisher.

## Footnotes

1
http://www.omicsmart.com
