# Supplementary material for: Peanut Rotation and Flooding Induce Rhizobacteriome Variation With Opposing Influences on the Growth and Medicinal Yield of Corydalis yanhusuo
Source: Front Plant Sci. 2022 Jan 7;12:779302. doi: 10.3389/fpls.2021.779302 (PMC8782247; doi:10.3389/fpls.2021.779302)
Supplement: Supplementary file 1 [file Data_Sheet_1.docx]

Supplementary Material

## Supplementary Figures


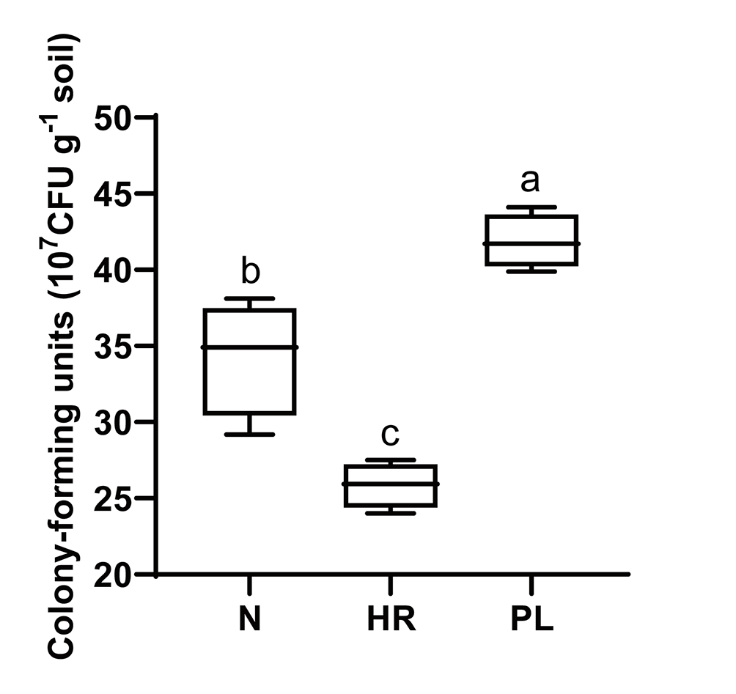


**Supplementary Figure 1.** Comparison of the quantity of culturable bacteria in rhizospheric soil of different field types in terms of their number of colony-forming units (CFUs). Values are the means of 4 duplicates with their SD. Differing lower-case letters indicate significant differences (*p* < 0.05). N, soil from the *C. yanhusuo* field without rotation; HR, soil from the *C. yanhusuo* field that had been flooded in July 2020; PL, soil from field under peanut–*C. yanhusuo* rotation.


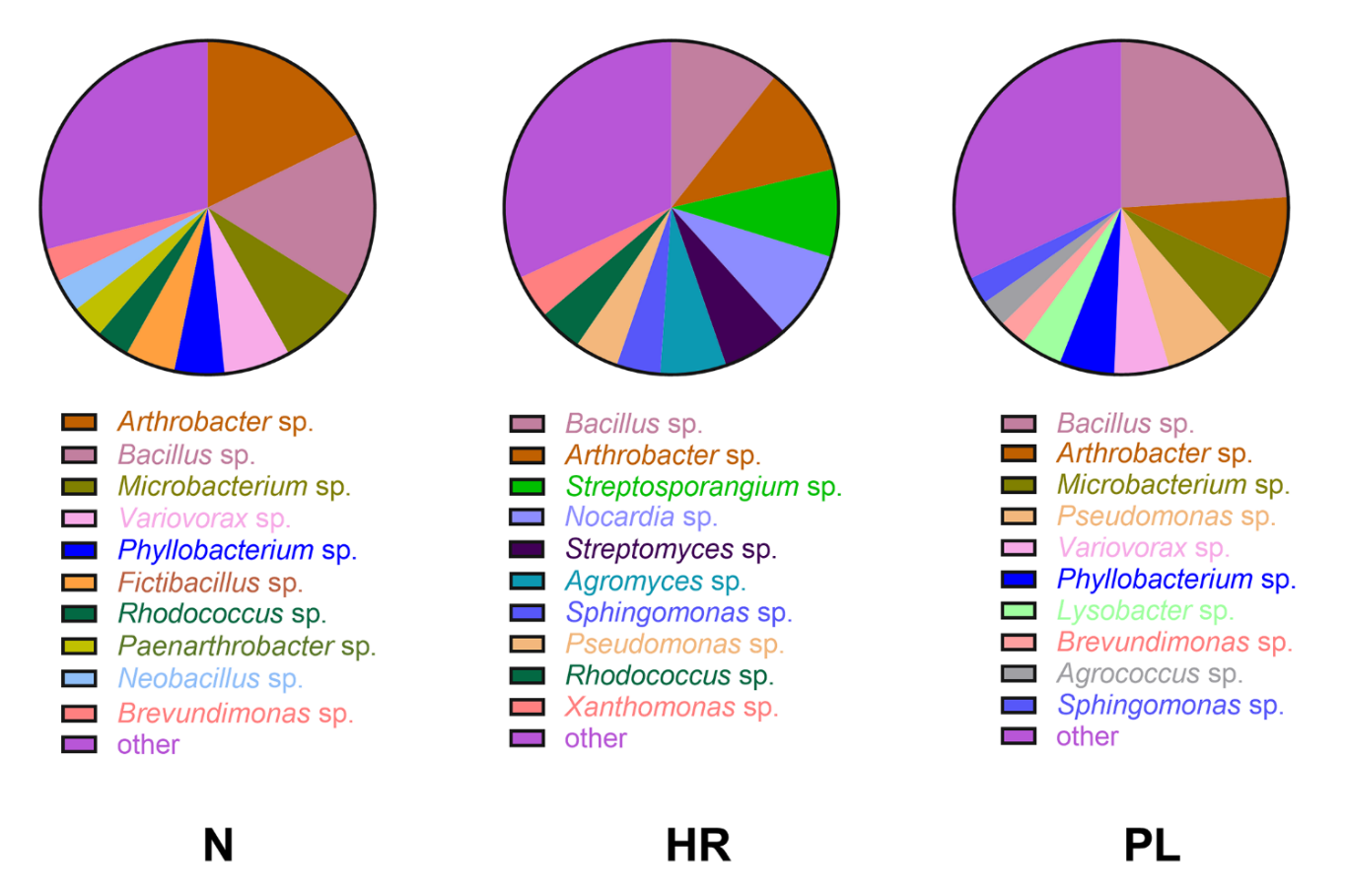


**Supplementary Figure 2.** Proportion of culturable bacteria in three different field types at the genus level. N, soil from the *C. yanhusuo* field without rotation; HR, soil from the *C. yanhusuo* field that had been flooded in July 2020; PL, soil from the field under peanut–*C. yanhusuo* rotation.


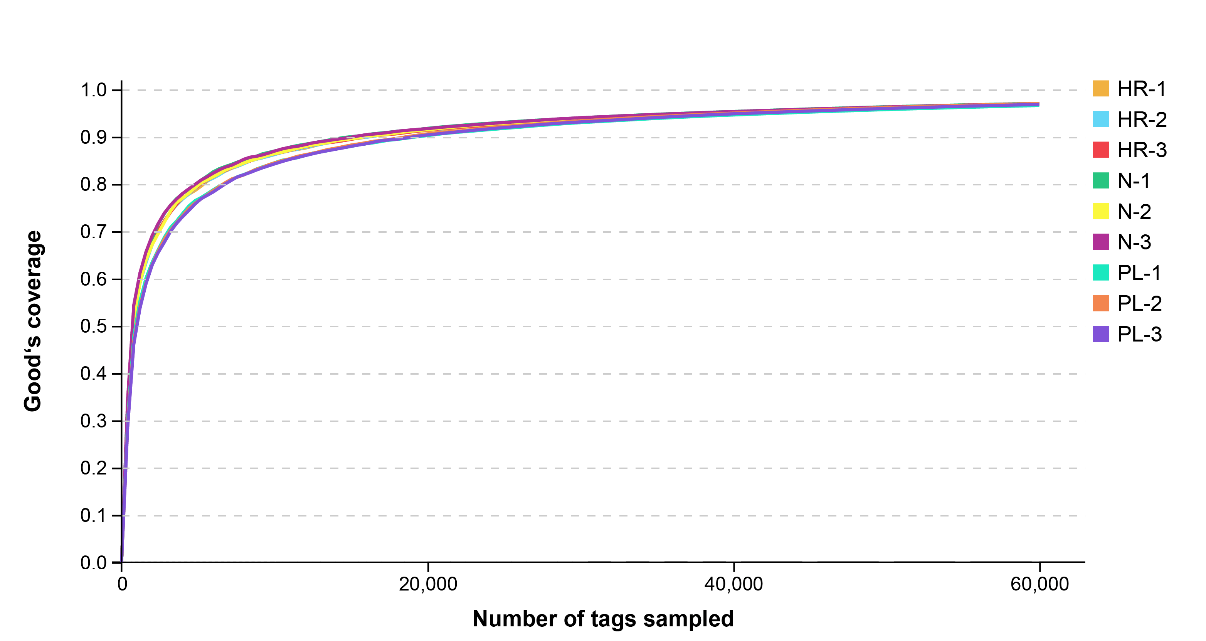


**Supplementary Figure 3.** Good’s coverage estimates for the nine rhizospheric soil samples of *C. yanhusuo.* N, soil from the *C. yanhusuo* field without rotation; HR, soil from the *C. yanhusuo* field that had been flooded in July 2020; PL, soil from the field under peanut–*C. yanhusuo* rotation.


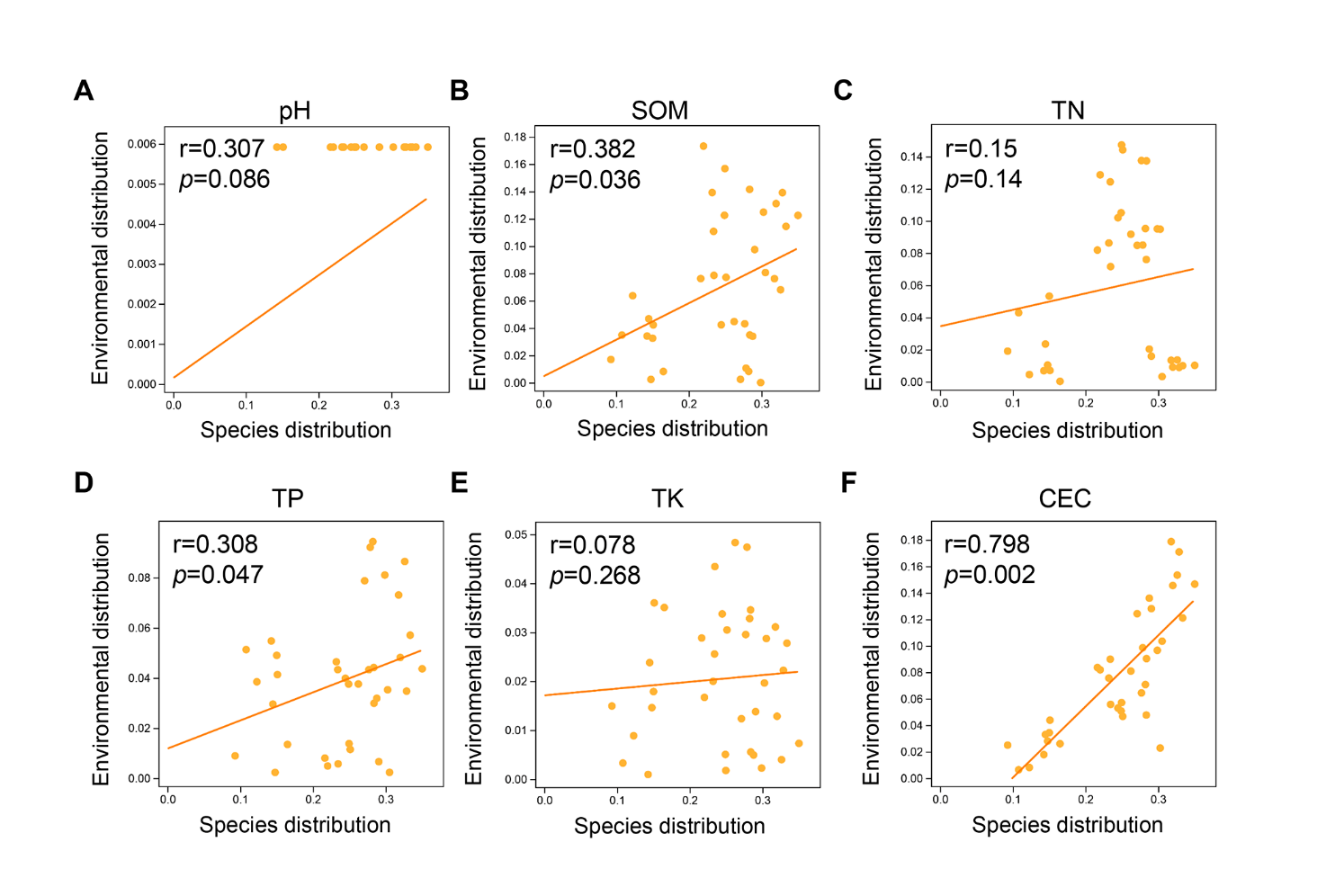


**Supplementary Figure 4.** Mantel test analysis of the bacterial community and measured environmental factors (soil physiochemical properties) at the genus level. pH **(A)**, SOM **(B)**, TN **(C)**, TP **(D**), TK **(E)**, and CEC **(F)**.


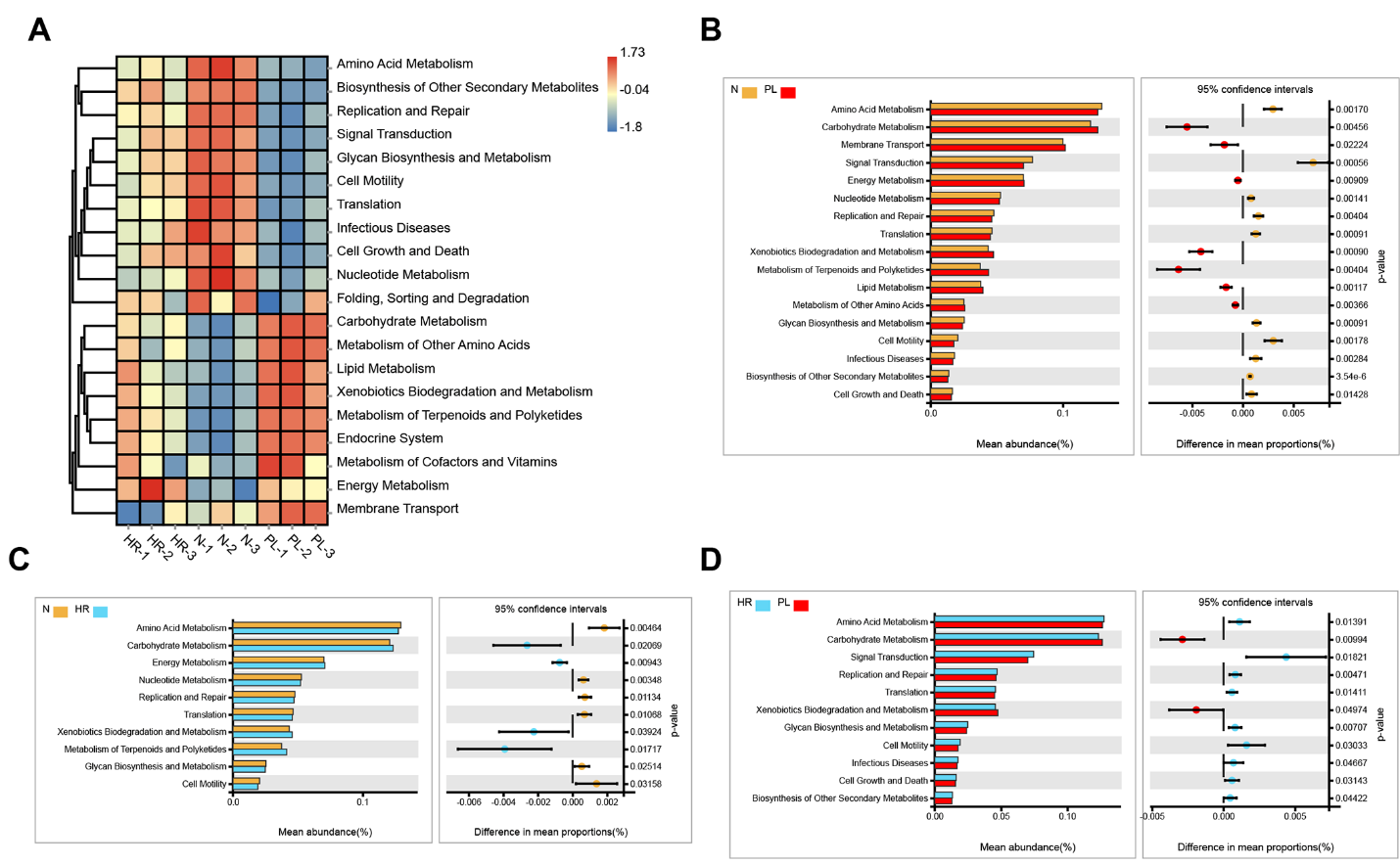


**Supplementary Figure 5.** Functional predictions of the rhizosphere bacteria from Tax4Fun. **(A)** Relative abundance (>1%) of potential functional pathways at level 2 in HR, N, and PL soils; Functional pathways found significantly abundant between **(B)** the N and PL soil (*p* < 0.05, Welch’s t-test), **(C)** the N and HR soil (*p* < 0.05, Welch’s t-test), and **(D)** the HR and PL soil (*p* < 0.05, Welch’s t-test). N, soil from the *C. yanhusuo* field without rotation; HR, soil from the *C. yanhusuo* field that had been flooded in July 2020; PL, soil from the field under peanut–*C. yanhusuo* rotation.

**
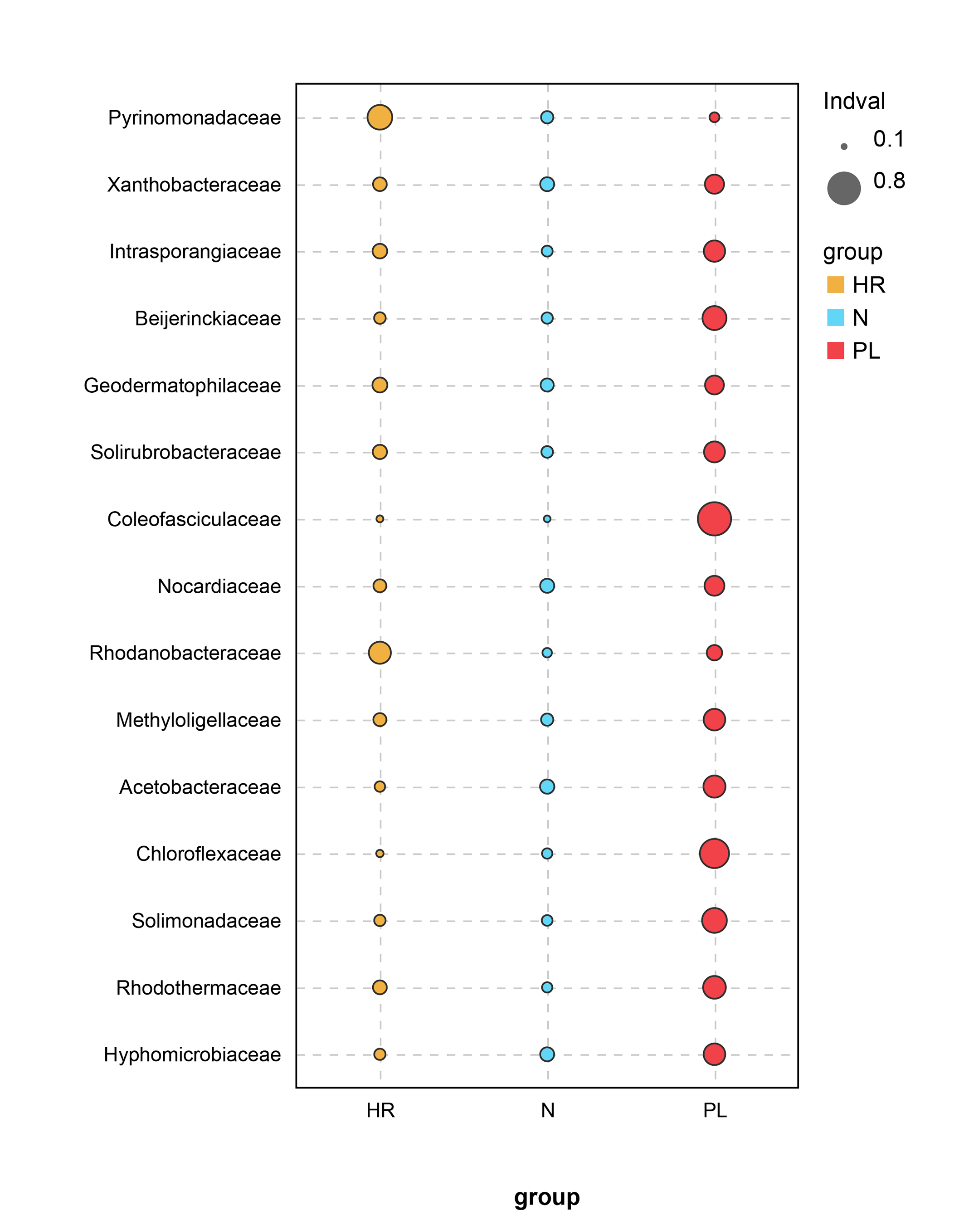
**

**Supplementary Figure 6.** Indval (indicator value) analysis of for samples from different fields at the family level. Relative abundance of the indicator taxa with indicator value index (*p* < 0.05). N, soil from the *C. yanhusuo* field without rotation; HR, soil from the *C. yanhusuo* field that had been flooded in July 2020; PL, soil from the field under peanut–*C. yanhusuo* rotation.

## Supplementary Tables

**Supplementary Table 1.** Tetrahydropalmatine content of *C. yanhusuo* (%).

| **Sample** | **Tetrahydropalmatine content (%)** |
| --- | --- |
| N | 0.2696±0.0263 a |
| HR | 0.3995±0.1069 a |
| PL | 0.3442±0.0958 a |

N, plants from the *C. yanhusuo* field without rotation; HR, plants from the *C. yanhusuo* field that had been flooded in July 2020; PL, plants from the field under peanut–*C. yanhusuo* rotation. The same lower-case lettering indicates no significant differences were found among these four samples (*p* < 0.05, one-way ANOVA).

**Supplementary Table 2.** Number of tags and operational taxonomic units (OTUs) obtained from each sample.

| **Sample** | **Total tags** | **OTUs** |
| --- | --- | --- |
| N-1 | 110643 | 5497 |
| N-2 | 109938 | 5692 |
| N-3 | 119906 | 5508 |
| HR-1 | 115301 | 5751 |
| HR-2 | 114136 | 5725 |
| HR-3 | 118982 | 5721 |
| PL-1 | 113496 | 6428 |
| PL-2 | 104909 | 6285 |
| PL-3 | 115100 | 6407 |

N, soil from the *C. yanhusuo* field without rotation; HR, soil the from *C. yanhusuo* field that had been flooded in July 2020; PL, soil from the field under peanut–*C. yanhusuo* rotation.

**Supplementary Table 3.** Sand, silt, and clay contents in the different soil samples.

| **Sample** | **Clay %** | **Silt %** | **Sand %** |
| --- | --- | --- | --- |
| N | 4.00±0.17 | 15.20±0.25 | 80.70±0.32 |
| HR | 5.70±0.12 | 25.10±0.38 | 69.20±0.46 |
| PL | 4.70±0.10 | 20.50±0.53 | 74.90±0.75 |

Values are the means of 3 duplicates with their SD. N, soil from the *C. yanhusuo* field without rotation; HR, soil the from *C. yanhusuo* field that had been flooded in July 2020; PL, soil from the field under peanut–*C. yanhusuo* rotation.
